# Photosynthetic production of enantioselective biocatalysts

**DOI:** 10.1186/s12934-015-0233-5

**Published:** 2015-04-15

**Authors:** Maik Bartsch, Sarah K Gassmeyer, Katharina Köninger, Kosuke Igarashi, Pasqual Liauw, Nina Dyczmons-Nowaczyk, Kenji Miyamoto, Marc M Nowaczyk, Robert Kourist

**Affiliations:** Junior Research Group for Microbial Biotechnology, Ruhr-Universität Bochum, Universitätsstr. 150, 44780 Bochum, Germany; Department of Biosciences and Informatics, Keio University, 3-14-1 Hiyoshi, Yokohama, 223-8522 Japan; Chair for Plant Biochemistry, Ruhr-Universität Bochum, Universitätsstr. 150, 44780 Bochum, Germany

**Keywords:** Enzyme expression, Sustainability, Photosynthesis, Enantioselectivity, Biocatalysis

## Abstract

**Background:**

Global resource depletion poses a dramatic threat to our society and creates a strong demand for alternative resources that do not compete with the production of food. Meeting this challenge requires a thorough rethinking of all steps of the value chain regarding their sustainability resource demand and the possibility to substitute current, petrol-based supply-chains with renewable resources. This regards also the production of catalysts for chemical synthesis. Phototrophic microorganisms have attracted considerable attention as a biomanufacturing platform for the sustainable production of chemicals and biofuels. They allow the direct utilization of carbon dioxide and do not compete with food production. Photosynthetic enzyme production of catalysts would be a sustainable supply of these important components of the biotechnological and chemical industries. This paper focuses on the usefulness of recombinant cyanobacteria for the photosynthetic expression of enantioselective catalysts. As a proof of concept, we used the cyanobacterium *Synechocystis* sp. PCC 6803 for the heterologous expression of two highly enantioselective enzymes.

**Results:**

We investigated the expression yield and the usefulness of cyanobacterial cell extracts for conducting stereoselective reactions. The cyanobacterial enzyme expression achieved protein yields of 3% of total soluble protein (%TSP) while the expression in *E. coli* yielded 6-8% TSP. Cell-free extracts from a recombinant strain expressing the recombinant esterase ST0071 from the thermophilic organism *Sulfolobus tokodai* ST0071 and arylmalonate decarboxylase from *Bordetella bronchiseptica* showed excellent enantioselectivity (>99% ee) and yield (>91%) in the desymmetrisation of prochiral malonates.

**Conclusions:**

We were able to present the proof-of-concept of photoautotrophic enzyme expression as a viable alternative to heterotrophic expression hosts. Our results show that the introduction of foreign genes is straightforward. Cell components from *Synechocystis* did not interfere with the stereoselective transformations, underlining the usability of photoautotrophic organisms for the production of enzymes. Given the considerable commercial value of recombinant biocatalysts, cyanobacterial enzyme expression has thus the potential to complement existing approaches to use phototrophic organisms for the production of chemicals and biofuels.

## Background

**Figure 1:**
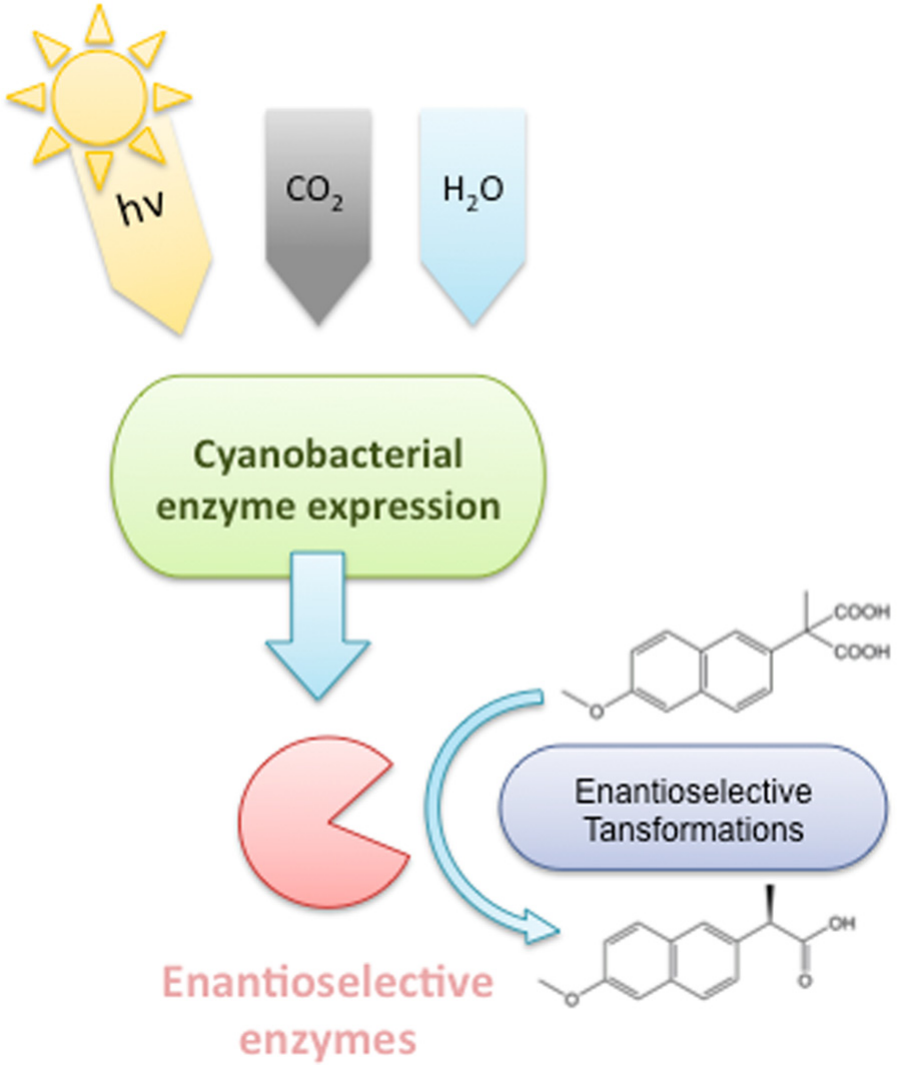
Cyanobacterial enzyme expression of enantioselective enzymes is a sustainable source of biocatalysts for the chemical industry.

Global resource depletion poses a dramatic threat to our society. Fossil-based resources will run out in the next decades, and alternative resources that do not compete with the production of food have to be identified. Meeting this challenge requires a thorough rethinking of all steps of the value chain, particularly regarding their resource demand and the possibility to substitute current, petrol-based supply-chains by renewable resources. This also regards the source of catalysts for the transformation of chemicals. Because biocatalysts function under very mild reaction conditions, they serve as very sustainable catalysts [1]. Their high selectivity makes it possible to shorten reaction routes and has led to numerous applications for the environmentally friendly synthesis of fine chemicals and pharmaceuticals [2]. The market for industrial enzymes grew nearly with double-digit pace from 2003 - in 2009 it reached $5.1 billion [3]. Enzyme production has relied so far mostly on heterotrophic expression systems that require substantial amounts of agriculturally produced nutrients. Direct utilization of photons by photoautotrophic enzyme production (Figure 1) allows the production of catalysts from little more than carbon dioxide, light and water [4]. Photoautotrophic microorganisms have the ability to utilize 15% of absorbed light for biomass formation [5]. Given the large amount of produced enzymes, photoautotrophic enzyme production has a very large potential to reduce the demand for reduced carbon sources as growth media, which in turn leads to considerably savings of fossil resources needed for the agricultural production of these media. Synthetic biology has emerged as a successful strategy for the expansion of the product spectrum and has already achieved the synthesis of 17 platform chemicals like 1-butanol and ethyle in phototrophic microorganisms [6,7]. Cyanobacterial catalyst production would be a step further towards a phototrophic biorefinery.

Wild-type cyanobacteria have shown high selectivity in whole-cell biotransformation, where the abundant supply of reduction equivalents from photosynthesis represents an additional advantage over heterotrophic systems [8]. Recent works show the feasibility and cost-effectiveness of the expression of therapeutic proteins and industrial enzymes such as xylanases and phytases in microalgae. Typical yields are in the range of 0.1-3% of the total soluble protein, but also yields up to 21% TSP have been achieved [4]. In contrast to eukaryotic microalgae [9], *Synechocystis* sp*.* PCC 6803 (*Synechocystis*) and other cyanobacteria are easy to manipulate [10,11], which makes the introduction of foreign genes straightforward. Cyanobacteria have been successfully cultivated in closed bioreactors and open ponds at low cost. The absence of a reduced carbon source also reduces the risk of contamination by bacteria or fungi. This simplifies the scaling significantly. Moreover, existing approaches for the production of biofuels and chemicals created a sophisticated technology for the large-scale cultivation of photoautotrophic bacteria. Photoautotrophic enzyme production thus has a high potential for biocatalytic applications. However, to our knowledge, cyanobacterial cell-extracts have not been applied for *in vitro* biocatalysis so far.

As a proof of concept, we applied cyanobacterial cell-extracts harbouring recombinant enzymes for the desymmetrization of malonic acids, a widely used reaction for the production of optically pure building blocks and pharmaceuticals [12,13]. Thermostable esterase ST0071 from the thermophilic archaeon *Sulfolobus tokodaii* shows high enantioselectivity in the desymmetrization of malonic acid diesters [14,15]. As ST0071 has inverse enantiopreference to porcine liver esterase, it complements this widely applied enzyme as an enantioselective catalyst [14]. Interestingly, ST0071 also possesses a promiscuitive, stereoslective decarboxylase activity [15]. While ST0071 produces optically active half esters, arylmalonate decarboxylase (AMDase) from *Bordetella bronchiseptica* catalyses the conversion of prochiral malonic acids to enantiomerically pure arylaliphatic carboxylic acids.

## Results

### Establishment of expression system

**Figure 2:**
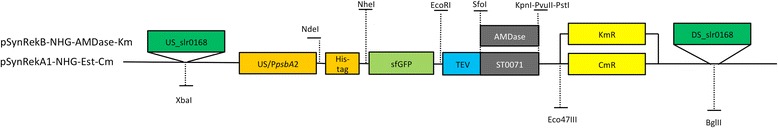
Plasmids for the site-directed genome integration of genes by homologous recombination.

**Figure 3:**
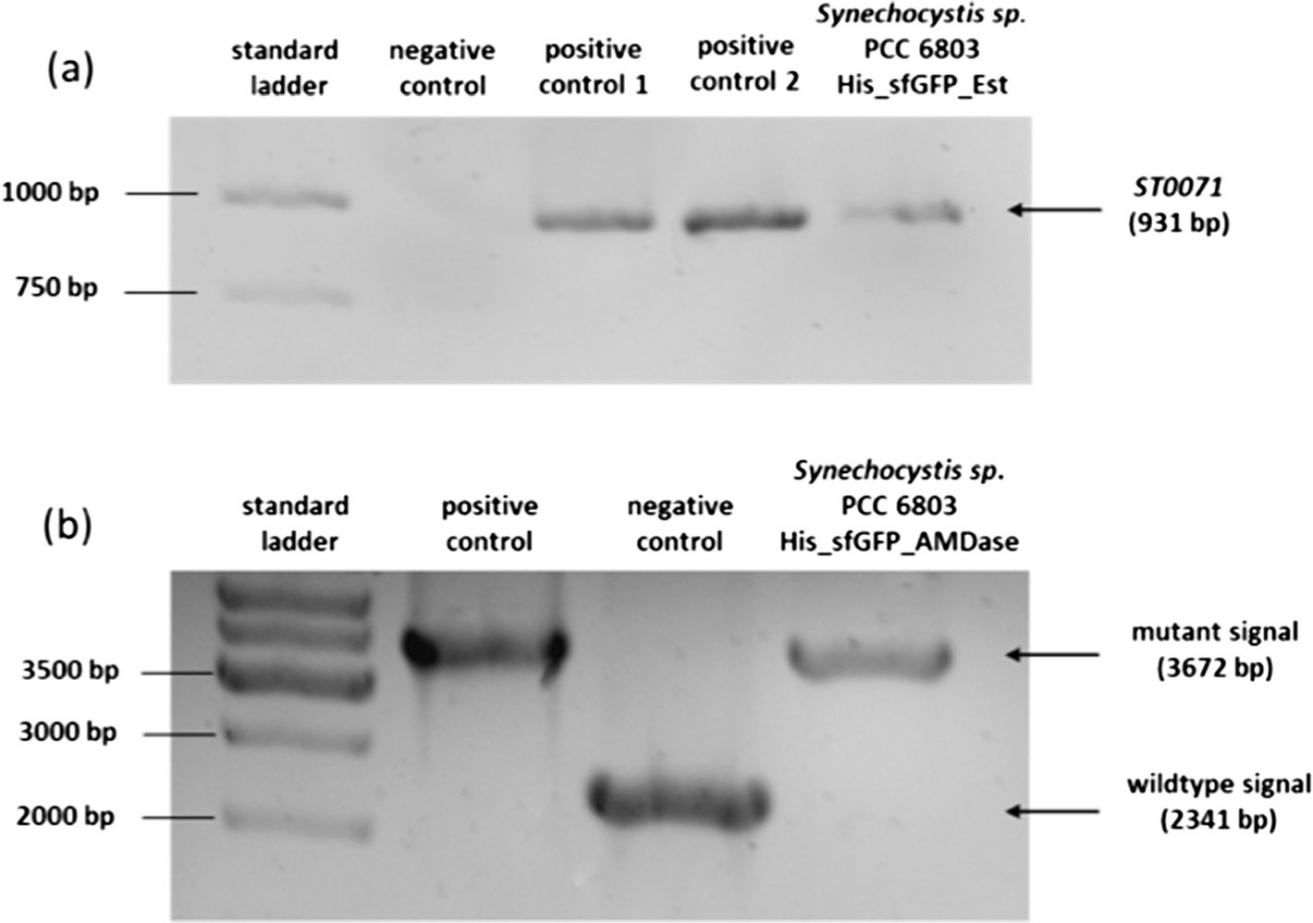
Confirmation of successful genome integration of **a**) the ST0071 gene and **b**) the AMDase gene by PCR.

The fact that *Synechocystis* contains up to 200 copies of its genome [16] and that foreign DNA is taken up spontaneously prompted us to use stable genome integration via homologues recombination for enzyme expression. The corresponding plasmids are compatible to a set of *E. coli* expression vectors that have been created in a previous study [17], thus allowing easy exchange of genes for expression in different host organisms. To facilitate the quantification of the enzymes in crude extracts, we created fusion proteins with super-folder GFP [18], an enhanced derivative of the well-known green fluorescent protein. The strong psbA2 promoter [19] was chosen to control expression of recombinant genes in cyanobacteria (Figure 2). As the construct contains a gene that mediates chloramphenicol resistance, raising the concentration of this antibiotic in a subsequent cultivation of 1-2 weeks increased the copy number of the gene by segregation. Thus, the whole process from cloning to segregation took 4-6 weeks. PCR experiments confirmed the successful integration of the genes ST0071 and AMDase into the *Synechocystis* genome (Figure 3).

**Figure 4:**
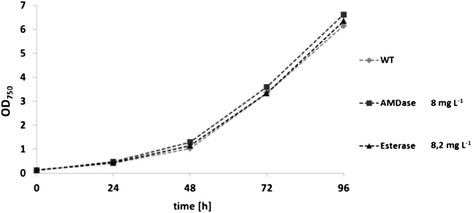
Cultivation in 5 L scale of *Synechocystis* wild type and recombinant strains with protein yields after 96 h.

**Figure 5:**
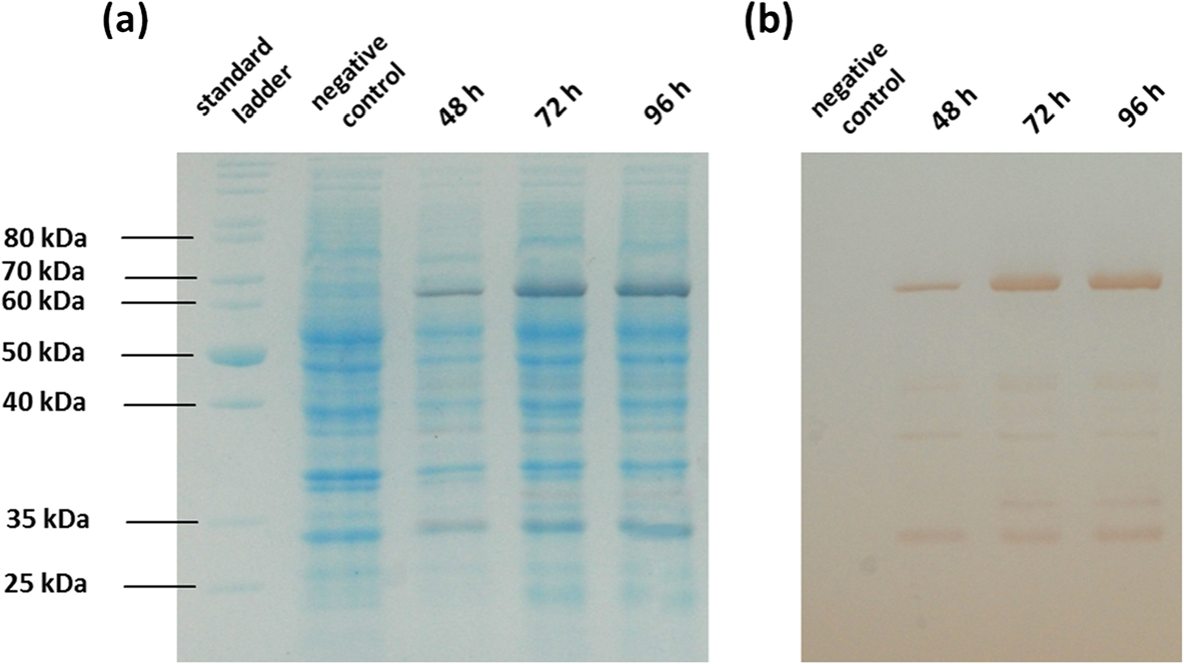
Functional expression of esterase ST0071 in *Synechocystis sp.* 6803. (**a**) SDS-PAGE of a functional expression of the sfGFP-fusion protein of esterase ST0071 (64 kD) in *Synechocystis* sp. PCC 6803 (**b**) Selective activity staining using α-naphthylacetate and Fast Red [29]. Cultures were grown in BG-11 media [28] in shake flasks and tubes at 30°C under a light strength of 300 μmol photons m^-2^ s^-1^ under an atmosphere containing 5% of CO_2_.

The *Synechocystis* strain bearing the recombinant gene ST0071 was cultivated in 4 L scale under constant ventilation with CO_2_ enriched air (5% CO_2_) using a light intensity of 300 μmol of photons m^-2^ s^-1^. Expression of the recombinant enzyme did not impair the growth rate (Figure 4). After four days of cultivation, cells were disrupted using glass beads. Enzyme concentration in crude extracts was determined by GFP fluorescence based on addition of a GFP standard (standard addition method). Esterase expression was visualised by activity staining (Figure 5) quantified by measuring the activity in the hydrolysis of p-nitrophenyl butyrate. Cyanobacterial cell extracts showed some background hydrolytic activity, which could be suppressed by a heat shock of 20 min. at 70°C. The light-inducible promoter psbA2 has been reported to increase enzyme expression at cultivation under highlight conditions [20]. Indeed, changing the light intensity from 60 μmol photons m^-2^ s^-1^ to 300 μmol m^-2^ s^-1^ increased the activity from 70 U g_CDW_^-1^ to 108 U g_CDW_^-1^. Under best conditions, determination of the protein concentration of the esterase in the cell-free extract showed 8.2 mg L^-1^ or 3.9% of total soluble protein (TSP). This means that the capacity of *Synechocystis* for the production of heterologous proteins is about two thirds of that of *E. coli* with 6.4% TSP (Table 1). This is a comparatively high expression yield in a phototrophic microorganism [4].

**Table 1:** **Comparison of the functional expression of sfGFP-Est ST0071 in** ***Synechocystis*** **sp. PCC 6803 and** ***E. coli***

	**Volumetric activity** ^**[1]**^	**Protein yield** ^**[2]**^	**Specific activity**	**Space time yield**	**Specific activity**
	**U L** ^**-1**^	**mg L** ^**-1**^	**U mg** ^**-1**^	**U L** ^**-1**^ **h** ^**-1**^	**U** ^**●**^ **g**_**CDW**_^**-1**^
**Synechocystis USpsbA2 Est**	411 ± 19	8.2 ± 2	50	4.3	108
**E. coli pRSET6a His_sfGFP_Est**	1639 ± 69	27.5 ± 4.4	60	273	441

^[1]^Determined in the hydrolysis of *para*-nitrophenyl butyrate; ^[2]^determined by standard-addition-method.

The resulting specific activity of 50 U mg^-1^ in cell free extracts of *Synechocystis* is in excellent agreement with the specific activity of 60 U mg^-1^ after cultivation in *E. coli*. Given the lower cell-densities that can be achieved with cyanobacteria in comparison to *E. coli*, we were pleased to find a volumetric activity of or 411 U L^-1^, which is 25% of the unit yield of the heterotrophic expression. Despite this encouraging result, the low growth rate of the cyanobacteria is still a considerable bottleneck. A cultivation batch of *Synechocystis* takes about 4 days, while cultivation in *E. coli* can be performed within 6 hours. The slow growth results in a relative space-time yield of only 1.6%.

**Figure 6:**
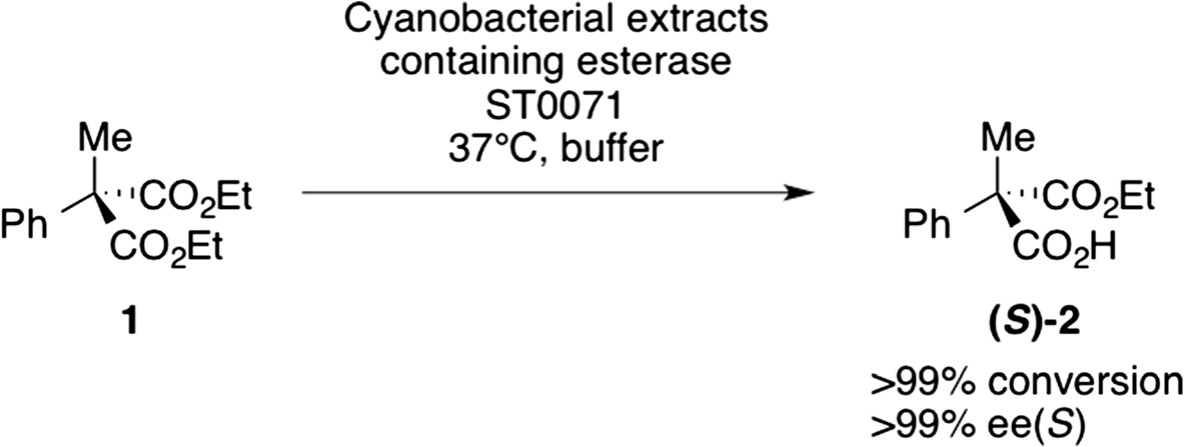
Esterase-catalysed desymmetrization of a prochiral malonic acid ester. Cell lysate from a cultivation *Synechocystis* sp. sfGFP-ST0071 cell lysate (500 μL of a 750 mL cultivation, OD 6.5) was added to a solution of 5 mM phenyl methyl malonate diethyl ester 1 and incubated for 48 h at 37°C. The reaction was stopped by addition of HCl (200 μL, 2 M), extracted twice with methyl *tert*-butyl ester. The optical purity of (S)-2 was determined using by chiral HPLC.

In order to investigate the effectiveness of the cyanobacterial expression system for biocatalytic reactions, we applied crude extracts bearing thermostable esterase ST0071 for the desymmetrization of phenyl methyl diethyl malonate **1** (Figure 6), esterase ST0071 showed a remarkably high selectivity and produced optically pure (*S*)-halfester with 99%ee(*S*) and >99% conversion (Table 2). The high selectivity of 99%ee(*S*) was also confirmed using His-tag purified enzyme from the *Synechocystis* cultivation. A control experiment using a strain without the esterase did not show any measurable activity in the hydrolysis of **1**, and heat treatment of the cell-free extract was not necessary.

**Table 2:** **Biocatalysis experiments using photoautotrophically produced enzymes**

**Biocatalyst**	**Substrate**	**Product**	**Time**	**Conversion**	**Enantiomeric excess**
			**[h]**	**[%]**	**[%]**
**Est ST0071 (cell-free-extract)**	**1a**	(*S*)-**2a**	180	>99%^[a]^	>99%^[a]^
**AMDase (cell-free-extract)**	**3b**	(*R*)-**4b**	2	>99%^[b]^	>99%^[b]^
**AMDase (cell-free-extract)**	**3c**	(*R*)-**4c**	2	>99%^[b]^	99%^[b]^

^[a]^Determined by chiral HPLC; ^[b]^Determined by chiral GC.

**Figure 7:**
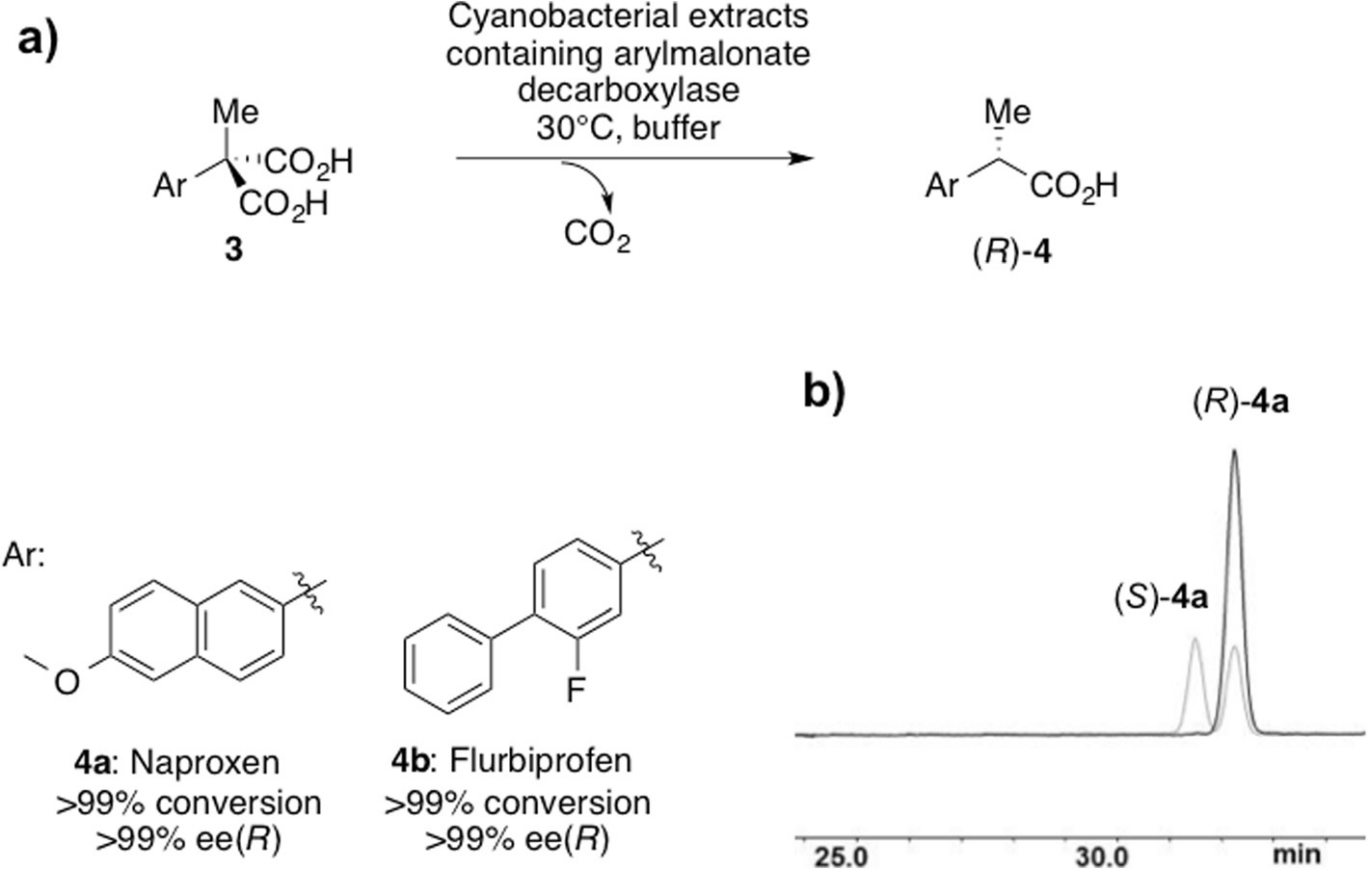
Arylmalonate decarboxylase-catalyzed desymmetrization of prochiral malonic acids. **a**) Desymmetrization of prochiral malonic acids 3a and 4b at 30°C using cell lysates from a cultivation of *Synechocystis* sp. sfGFP-AMDase. **b**) GC-chromatogram of optically pure (*R*)-4a (grey) and a racemic standard (light grey).

Arylmalonate decarboxylase was introduced with a similar strategy as fusion protein with sfGFP into the neutral site slr1608 of the *Synechocystis* genome. After confirmation of complete segregation, the expression of the AMDase under highlight conditions yielded 335 U L^-1^ in the decarboxylation of phenylmalonate and a protein concentration of 8.0 mg L^-1^. Similar to the esterase expression, this is about one fourth of the yield in *E. coli* (33.2 mg L^-1^) and corresponded to 3.0% TSP in comparison to 7.8% TSP in *E. coli*. His-tag purification yielded 5.7 mg L^-1^, which is a purification yield of 68%. Cell free extracts containing AMDase converted prochiral malonates to optically pure naproxen **4a** and flurbiprofen **4b** with very high enantioselectivity (Figure 7, Table 2). The high selectivity towards **3a** and **3b** was confirmed using purified enzyme. In a control reaction, crude extracts without AMDase did not show any decarboxylating activity. The example of AMDase shows that the approach is thus applicable also with enzymes from mesophilic organisms. In a preparative-scale reaction batch, *Synechocystis* cell-free extract produced 76 mg optically pure (*R*)-naproxen in very good yield and excellent optical purity (91%, >99% ee).

## Discussion

Our findings unveil the proof-of-concept in using cyanobacteria for producing enantioselective enzymes using CO_2_, water and light. The results show that *Synechocystis* is capable to produce recombinant enzymes in concentrations of 8-9 mg L^-1^ and 3-4% TSP, which is significantly higher than enzyme yields reported for microalgae [4]. It is an important observation that cyanobacterial cell components do not interfere with the enantioselective reactions, which is an important prerequisite for commercial applications. For practical applications it should also be emphasized that the manipulation of *Synechocystis* is straightforward and can be conducted in 4-6 weeks.

Compared to *E. coli*, the cyanobacterial enzyme expression achieves a specific activity of 25%. For an initial experiment in comparison to a well-established expression system, this is a very encouraging result, due to the inherent sustainability to produce the biomass directly from carbon dioxide and light instead from media that require the detour of agricultural production.

Volumetric enzyme yields of up to 25% of *E. coli* show that the water demand, a frequent concern in the application of photoautotrophic organisms, will not be much higher than with heterotrophic organisms. However, at this stage of engineering, the slow growth and the demand for freshwater hinder potential commercial applications. Continuous cultivation systems have a potential to increase space-time-yields. For the time being, *Synechocystis* sp. offers the advantage of the availability of suitable genetic tools for the introduction and functional expression of heterologous genes. The development of tunable promoters and other tools is undergoing a rapid progress [10,11]. Fast-growing and salt-water tolerant strains [6,7] are expected to alleviate the slow growth in a longer perspective and will pave the way for a cyanobacterial biomanufacturing platform using waste-water or seawater. The demand for these strains has been recognized, and on-going research [10,11] will make these strains amenable for genetic manipulation and facilitate their application for recombinant enzyme production.

Cell-free-extracts from *Synechocystis* contain similar amounts of biocatalysts than *E. coli*. This is significantly different to approaches that aim to utilize *Synechocystis* for chemical production, where yields are several magnitudes lower than in *E. coli* or *Synechocystis*. Cyanobacteria have been demonstrated to be very proficient whole-cell biocatalysts for the biotransformation of organic compounds [8]. Our results show that recombinant cyanobacterial strains are highly useful expression systems for the environmentally-friendly production of enzymes.

The excellent enantioselectivities in the synthesis of the optically pure phenyl methyl malonate ethyl ester (*S*)- **2a** and the optically pure profens naproxen and flurbiprofen demonstrate the feasibility of the application of cyanobacterial cell-free extracts for catalytic purposes. The cyanobacterial components do not interfere with the reaction, which is an important prerquisite for commercial applications. Expansion of this approach to the recombinant expression of oxidoreductases will be particularly interesting, as cyanobacteria can also supply NADPH as cofactor, resulting in very proficient whole-cell biocatalysts for the biotransformation of organic compounds [8]. The successful production of enzymes in phototrophic organisms is an important step towards a photoautotrophic biorefinery, in which cyanobacteria and microalgae convert carbon dioxide, light and water to useful chemicals such as bio-hydrogen [21], terpenes [22], carotinoids [19] and fatty acids [23]. Photoautotrophic enzyme production will be a sustainable strategy to provide biocatalysts for this visionary biorefinery. Enzyme production can use the infrastructure and the facilities of the main process, which creates further synergistic effects. A prominent example for the *on-site* production of biocatalysts in industrial scale is the synthesis of cellulolytic enzymes in brewer’s yeast [24-26].

## Conclusions

We were able to present the proof-of-concept of photoautotrophic enzyme expression as a viable alternative to heterotrophic expression hosts. Cell-free extracts from *Synechocystis* were successfully applied as catalysts for the asymmetric synthesis in high conversion and excellent enantioselectivity. Our results show that the introduction of foreign genes is straightforward and that cell components do not interfere with enzymatic reactions. Photoautotrophic enzyme expression achieves yields of about 25% of the established heterotrophic system *E. coli*. Nevertheless, cyanobacterial biomass stems directly from photosynthesis, while heterotrophic organisms require the detour of agricultural plant cultivation, harvest and processing. The great demand of an enzyme market of $5.1 billion [3] underlines the great potential of photosynthetic biocatalyst production for energy and fossil resources savings.

## Methods

### General

All chemicals were purchased from Sigma-Aldrich (Steinheim, Germany). Arylmalonic acids **3b**, **3c** and **3d** were kindly provided by Chiracon GmbH (Teltow, Germany). Fluorescence and absorption were measured with a FLUOstar Omega Fluorimeter (BMG Labtech GmbH Ortenberg, Germany) DNA samples were sequenced by the DNA sequencing service of the department of biochemistry at Ruhr-University Bochum. Kits for ligation, DNA isolation and restriction were purchased at Fermentas (St. Leon-Rot, Germany). Enzyme affinity purification was done using a 1 mL His Pure Ni-NTA column (Pierce Biotechnology Rockford, USA). Chiral GC analyses were carried out using the chiral column FS-Hydrodex-β-6TBDM [heptakis-(2,3-di-O-methyl-6-O-*t*-butyldimethylsilyl)-β-cyclodextrin] (Macherey Nagel, Germany) on a GC-FID-2010 (Shimadzu, Japan). Retention times for **2b** (160°C) were 31.5 min (*S*) and 32.3 min (*R*) for **2c** (170°C) 12.9 min (*S*) and 14.4 min (*R*). The elution order was identified using commercial (*S*)-naproxen (TCI Chemicals, Japan). Chiral HPLC analyses were carried out using the column CHIRALCEL OD-H (Daicel, Osaka, Japan) on a L-7110 HPLC device (Hitachi High Tech, Tokyo, Japan) using n-hexane/i-propanol/trifluor acetic acid (98:2:0.5). With a flow rate of 0.5 mL, the two enantiomers of phenyl methyl malonic acid ethyl ester **2a** eluted with 55 min (*S*) and 65 min (*R*).

### Expression of GFP-esterase and GFP-AMDase fusion proteins in *E. coli*

The vector pET100 ST0071 containing the native gene of esterase ST0071 [12] and pBAD_AMDase containing a codon optimized gene of AMDase from *Bordetella bronchiseptica* pBAD[22] were kindly provided by Kenji Miyamoto, Keio University (Japan). For overexpression of both proteins as fusion proteins with an N-terminal His6-sfGFP tag, genes were amplified by PCR using the oligonucleotides GGCGCCGCTAGCATAGACCCTAAAATTA or GCGAATTCGCG-GCGCGATGGGCCAAATGCAACAGG (forward primer), GATG-GTACCTTATTTGTAGAGCTCTTTTCC or GCCTGCAGTTAGCT-GCCACCGGACTCAT (reverse primer) and cloned into the pNHIS-GFP-TEV plasmid [19] via the SfoI and KpnI restriction enzyme sites. The resulting plasmids pNHG-Est and pNHG-Amd were used for transformation of *E. coli* overexpression C43 cell (Lucigen) and protein expression was performed according to literature [17]. For the determination of dry cell weight (DCW), 15 mL of the culture supernatant was added in triplicate to pre-weighed reaction tubes and centrifuged for 4 min at 13000 rpm and 4°C. After a subsequent washing step with 0.9% (w/v) NaCl, cell pellets were dried until constant mass at 60°C for at least 4 days.

### Construction of plasmids for expression of GFP fusion proteins in synechocystis

For heterologous expression of the GFP-esterase fusion in *Synechocystis* under the control of the strong psbA2 promoter [19], the plasmid pSynRekA1-NHG-Est-Cm was constructed, that allows replacement of the native psbA2 gene by homologous recombination. A 500 bp upstream region of psbA2 gene was amplified by PCR with the primer pair GATCTAGACAGAATCCTTGCCCAGATGC and CGTTGTCATATGGTTATAATTCC and cloned into the pNHG-Est plasmid via the XbaI and NdeI restriction enzyme sites. In the last step an antibiotic resistance cassette that is conferring resistance to chloramphenicol, was inserted into the BamHI site of the plasmid.

For expression of the GFP-AMDase fusion in *Synechocystis*, the plasmid pSynRekB-NHG-AMDase-Km was generated. This allows integration at the neutral site of slr0168 [27] and expression under the control of the psbA2 promoter. The 800 bp upstream region of slr0168 was amplified by PCR with the primer pair TCTAGAGAGTTATTGGCGATCGAAGC and CTAGCTTAGGGGGTGTATTGAATAGTCATAG and cloned into the XbaI site of an intermediate pSynRekA-NHG-Est construct without the Cm cassette. The 800 bp downstream region of slr0168 was amplified by PCR with the primer pair GGATCCAGCGCTTGGCATCAGCCACAGCACAAAC and AGATCTCACCACCTTGGGCTTGATGC and cloned into the BglII site. In the next step a kanamycin resistance cassette was cloned into an Eco47III site that was introduced by the forward primer used for PCR amplification of the downstream fragment. Finally, the coding sequence of the Esterase was replaced by that of the AMDase via the SfoI and KpnI sites.

### Transformation of *Synechocystis*

Wild type cells of 1 mL culture (OD750 = 2) were harvested by centrifugation and resuspended in 100 μl fresh BG11 media. Plasmid DNA was added to a final concentration of 0.02 μg × mL^-1^ and cells were incubated in darkness for 5 h at 30°C. After recovery treatment, transformants were incubated another 24 h at low light conditions (<60 μmol photons m^-2^ × s^-1^) and finally spread on BG-11 plates with the corresponding antibiotic chloramphenicol (5 μg × mL^-1^) or kanamycin (40 μg × mL^-1^). In order to promote full segregation of the mutant allele, the selective pressure was increased gradually to a final concentration of 50 μg × mL^-1^ (chloramphenicol) and 150 μg × mL^-1^ (kanamycin). Successful integration was confirmed by PCR.

### *Synechocystis* culture conditions and preparation of cell extracts

*Synechocystis* strains were cultivated in BG-11 media (see also the electronic supplementary information) [28] in shake flasks and tubes at 30°C under varying light conditions from 60 μmol photons m^-2^ s^-1^ up to 300 μmol photons m^-2^ s^-1^ under an atmosphere containing 5% of CO_2_. For functional expression of esterase and AMDase, the media were supplemented with chloramphenicol (50 μg mL^-1^) or kanamycine (150 μg mL^-1^), respectively.

For preparation of raw extracts, cells were harvested by centrifugation (20 min, 4°C, 8,000 rpm) and washed with buffer. Cell disruption was performed with a Precellis Homogenizer (PEQLAB Biotechnologie GmbH, Erlangen, Germany) in precooled 2 mL screw-cap tubes containing 750 μl glass beads (0.1-2 mm diameter) with two pulses of 30s with 6800 oscillations per minute. The supernatant was transferred to another tube and centrifuged in order to remove cell debris (67,000 g, 40 min. 4°C). After cell disruption by French press, this step can alternatively conducted with 10,000 g for 30 min. and 4°C. After SDS-gel electrophoresis, the gel was first activity-stained with α-naphthylacetate and Fast Red [29] followed by Coomassie brilliant blue staining.

### Fluorescence standard addition for protein determination

As cyanobacterial cell extracts showed considerable background fluorescence, standard addition was chosen to quantify sfGFP fusion proteins. Purified sfGFP fusion esterase or AMDase were added as standard directly to cyanobacterial crude extract. The optimal size of each addition was chosen generating a signal 1.5 to 3 times that of the unknown sample. using excitation-/emission-filters at 480-/520 nm, respectively. From at least 5 measurements, a line was extrapolated to zero fluorescence, and the endogenous concentration of analyte was determined from the point of intersection with the abscissa. The percentage of purified enzymes of the total soluble protein (TSP) was determined by relating these values to protein concentrations determined with Bradford reagent.

### Biocatalysis experiments

Esterase activity was determined in triplicates spectrophotometrically by hydrolysis of *p*-nitrophenyl acetate (10 mM in DMSO) in sodium phosphate buffer (10 mM, pH 7.4). *p*-Nitrophenol released was quantified at 410 nm (ε = 15.1*10^3^ M^-1^ cm^-1^). One Unit (U) of activity was defined as the amount of enzyme releasing 1 μmol *p*-nitrophenol per min under assay conditions. For desymmetrization experiments, cell lysate from a cultivation *Synechocystis* sp. sfGFP-ST0071 cell lysate (500 μL of a 750 mL cultivation, OD 6.5) was added to a solution of 5 mM phenyl methyl malonate diethyl ester 1 and incubated for 48 h at 37°C. The reaction was stopped by addition of HCl (200 μL, 2 M), extracted twice with methyl tert butyl ester. The optical purity of (S)-**2** was determined using a Chiracell OD-H column on an HPLC-device (Shimadzu, Japan) with a mixture of *n*-hexane (97%) and *iso*-propanol (3%) as mobile phase.

### Preparative scale AMDase-catalyzed asymmetrization

Synechocystis sp. sfGFP-AMDase cell lysate (35 mL of a 750 mL cultivation, OD 6.5) was added to a stirred solution of 5 mM 2-(6-ethoxy-2-naphthyl) malonic acid (100 mg, 0.36 mmol) in tris buffer (50 mM, 300 mM NaCl, pH 7.5) to a total volume of 73 mL, respectively. The reaction mixture was stirred for 4 hours at 30°C and stopped by addition of HCl (35 mL, 2 M). The reaction mixture was extracted three times with methyl tert-butyl ether (100 mL). The organic layers were combined and washed with brine (2 × 50 mL), and dried over anhydrous MgSO_4_. The solvent was removed under reduced pressure to yield optically pure (*R*)-2-(6-ethoxy-2-naphthyl) propionic acid ((*R*)-**4b**) as yellowish powder (76.2 mg, 0.33 mmol, 91.7%, >99% ee). 1H NMR: d = 1.59 (3H, d, CH3), 3.87 (1H, q, CH), 3.90 (3H, q, OCH3), 7.09, 7.71 (6H, m, Ar-H); 13C NMR: d = 18.0 (s), 45.1 (s), 55.3 (s), 105.5 (s), 119.0 (s), 126.1 (s), 127.3 (s), 128.8 (s), 129.3 (s), 133.8 (s), 134.9 (s), 157.7 (s), 179.6 (s).
